# Early management after self-poisoning with an organophosphorus or carbamate pesticide – a treatment protocol for junior doctors

**DOI:** 10.1186/cc2953

**Published:** 2004-09-22

**Authors:** Michael Eddleston, Andrew Dawson, Lakshman Karalliedde, Wasantha Dissanayake, Ariyasena Hittarage, Shifa Azher, Nick A Buckley

**Affiliations:** 1 South Asian Clinical Toxicology Research Collaboration, Centre for Tropical Medicine, Nuffield Department of Clinical Medicine, University of Oxford, UK; 2 Department of Clinical Medicine, University of Colombo, Sri Lanka; 3 Department of Pharmacology, University of Newcastle, Australia; 4 Department of Clinical Medicine, University of Peradeniya, Sri Lanka; 5 Medical Toxicology Unit, Guy's and St Thomas's Hospitals, London, UK; 6 Anuradhapura General Hospital, North Central Province, Sri Lanka; 7 Polonnaruwa General Hospital, North Central Province, Sri Lanka; 8 Department of Clinical Pharmacology and Toxicology, Canberra Clinical School, ACT, Australia

**Keywords:** atropine, carbamate, management, organophosphate, pesticides

## Abstract

Severe organophosphorus or carbamate pesticide poisoning is an important clinical problem in many countries of the world. Unfortunately, little clinical research has been performed and little evidence exists with which to determine best therapy. A cohort study of acute pesticide poisoned patients was established in Sri Lanka during 2002; so far, more than 2000 pesticide poisoned patients have been treated. A protocol for the early management of severely ill, unconscious organophosphorus/carbamate-poisoned patients was developed for use by newly qualified doctors. It concentrates on the early stabilisation of patients and the individualised administration of atropine. We present it here as a guide for junior doctors in rural parts of the developing world who see the majority of such patients and as a working model around which to base research to improve patient outcome. Improved management of pesticide poisoning will result in a reduced number of suicides globally.

## Introduction

Pesticide self-poisoning is a major clinical problem in many parts of the world [1,2], probably killing about 300,000 people every year [3,4]. Although most deaths occur in rural areas of the developing world [2], pesticide poisoning is also a problem in industrialized countries, where it may account for a significant proportion of the deaths from self-poisoning that do occur [5,6].

The case fatality for self-poisoning in the developing world is commonly 10–20%, but for particular pesticides it may be as high as 50–70% [2]. This contrasts with the less than 0.3% case fatality ratio normally found for self-poisoning from all causes in Western countries. The causes of the high case fatality are multifactorial but include the high toxicity of locally available poisons, difficulties in transporting patients across long distances to hospital, the paucity of health care workers compared with the large numbers of patients, and the lack of facilities, antidotes, and training for the management of pesticide-poisoned patients [2,4].

The problem is compounded by a lack of proven interventions with which to develop treatment protocols. In 2002 we set up a cohort study in the North Central Province of Sri Lanka that sought to follow 10,000 acutely self-poisoned patients prospectively. So far, over 6000 patients have been recruited, of whom more than 3000 have ingested pesticides. All patients are rapidly resuscitated on admission to hospital and stabilised according to a standard protocol.

Basic pharmacology and animal work suggests that early antagonism of pesticide toxicity should be associated with better outcomes [7,8]. Although there are few studies on the subject, there is some evidence that patients in the developing world often die soon after admission ([9], and CGS Rao, unpublished data). The rapid and effective stabilisation and treatment of pesticide-poisoned patients on their admission should reduce the number of early deaths, improve the prognosis for surviving patients over the next few days, and reduce the number and severity of long-term sequelae.

## Organophosphorus and carbamate pesticide poisioning

This paper presents the protocol that we use to treat organophosphorus (OP)-poisoned or carbamate-poisoned patients on admission, based on our clinical experience and the best available evidence (see Additional file 1 ). It focuses on intentional ingestion of pesticides because such patients are more often severely poisoned than those with accidental or occupational exposure. We have not used any of the published severity poisoning scales because none have been independently validated. More importantly, pesticide-poisoned patients are unstable and a mildly poisoned patient can rapidly become very ill. An initial severity score suggesting a mild poisoning might allow doctors to relax with unfortunate results, as recognised by the IPCS/EC/EAPCCT poison severity score, which is designed only to be used retrospectively [10].

## Poisoning with other pesticides

We concentrate here on OPs and carbamate pesticides because OPs in particular are responsible for most pesticide deaths across Asia [2,11-13]. In addition, careful administration of oxygen, atropine and mechanical ventilation offers the opportunity to make a significant difference in outcome. However, the protocol can be adapted for the resuscitation of patients poisoned with other pesticides. Readers are referred to textbooks of clinical toxicology for details of subsequent treatment.

## Initial assessment of the unconscious patient

Initial assessment involves checking airway, breathing and circulation. As part of this process, provide high-flow oxygen if available and ensure a patent airway through the placement of a Guedel airway or access.

Place the patient in the left lateral position, ideally in a head-down position, to reduce the risk of aspiration. Extension of the neck in this position helps to keep the airway patent.

Watch out for convulsions and treat with intravascular (IV) diazepam immediately if they do occur. Record a baseline Glasgow Coma Score to help with subsequent monitoring of the patient's condition. If available, affix a pulse oximeter.

## Does the patient require atropine? Recognition of OP/carbamate poisoning

Next, assess whether the patient requires atropine. Textbooks list many features of the cholinergic syndrome [14,15]. However, we use five in routine assessment: miosis, excessive sweating, poor air entry into the lungs due to bronchorrhoea and bronchospasm, bradycardia, and hypotension.

Severely OP- or carbamate-poisoned patients are typically covered with sweat, and have small pinpoint pupils and laboured breathing (often with marked bronchorrhoea and wheeze). The presence of pinpoint pupils and excessive sweat suggests that the patient has taken an OP or carbamate and requires atropine. The heart rate may be slowed, but normal or even fast heart rates are common.

If none of these signs are present, then the patient does not yet have clinical cholinergic poisoning and does not require atropine. However, it is possible that these signs will occur later, for example as a pro-poison (thion) OP is converted to the active oxon form, as a fat-soluble OP such as fenthion leaches out of fat stores into the blood, or if the patient has presented soon after the ingestion. Careful observation is required to look for the development of cholinergic signs.

## Loading with atropine and IV fluids

### Dose of atropine

For an unconscious patient, give atropine 1.8–3 mg (three to five 0.6 mg vials) rapidly IV into a fast-flowing IV drip. Although it is preferable that oxygen is given early to all ill patients, do not delay giving atropine if oxygen is unavailable. Because atropine dries secretions and reduces bronchospasm, its administration will improve patient oxygenation. There is no good evidence that giving atropine to a cyanosed patient causes harm.

Atropine takes only a few minutes to work. During the 5 min after atropine administration, record three other signs of cholinergic poisoning against which atropine dosing will be titrated (Table 1): (1) air entry into lungs; (2) blood pressure; (3) heart rate.

There is no need to do this before atropine is given, because pinpoint pupils and sweating in a region where these pesticides are common are sufficient to indicate OP/carbamate poisoning and trigger the decision to give atropine.

If the clinical presentation is not clear, administer atropine 0.6–1 mg. A marked increase in heart rate (more than 20–25 beats/min) and flushing of the skin suggest that the patient does not have significant cholinergic poisoning and further atropine is not required.

### Giving fluids

While waiting for the atropine to have effect, ensure that the two IV drips have been set up (one for fluid and drugs, the other for atropine). Give 500–1000 ml (10–20 ml/kg) of normal saline over 10–20 min.

### Assess whether enough atropine has been given – is the patient atropinised?

Three to five minutes after giving atropine, check the five markers of cholinergic poisoning (Table 2). Mark them on an OP/carbamate observation sheet (Table 1). A uniform improvement in most of the five parameters is required, not improvements in just one. However, the most important parameters are air entry on chest auscultation, heart rate, and blood pressure.

Pupil dilatation is sometimes delayed. Because patients do not die from constricted pupils, and the other parameters may improve more rapidly, it is reasonable to wait for the pupils to dilate. Check frequently and carefully that the other parameters are improving.

When all the parameters are satisfactory, the patient has received enough atropine and is 'atropinised'.

### Continuation of bolus atropine loading to reach atropinisation

If after 3–5 min a consistent improvement across the five parameters has not occurred, then more atropine is required. Double the dose, and continue to double each time that there is no response [16,17] (Table 1). Do not simply repeat the initial dose of atropine. Some patients need tens or hundreds of mg of atropine, so repeating 3 mg doses will mean that it may take hours to give sufficient atropine [16]. Severely ill patients will be dead by this point – atropinise the patient as quickly as possible.

Beware of pupils that do not dilate because pesticide has been splashed into them directly, and lung crepitations that are due to aspiration of the pesticide rather than the systemic effects of the pesticide. Generalised wheeze may be a better sign of under-atropinization in a patient who has aspirated pesticide.

### Atropine treatment after atropinization

Once atropinised (with clear lungs, adequate heart rate [more than 80 beats/min] and blood pressure [more than 80 mmHg systolic with good urine output], dry skin, and pupils no longer pinpoint), set up an infusion using one of the two IV cannulae. This should keep the blood atropine concentration in the therapeutic range, reducing fluctuation compared with repeated bolus doses.

In the infusion, try giving 10–20% of the total amount of atropine that was required to load the patient every hour. If very large doses (more than 30 mg) were initially required, then less can be used. Larger doses may be required if oximes are not available. It is rare that an infusion rate greater than 3–5 mg/hour is necessary. Such high rates require frequent review and reduction as necessary.

## Observation of the patient

Review the patient and assess the five parameters every 15 min or so to see whether the atropine infusion rate is adequate. As atropinisation is lost, with for example recurrence of bronchospasm or bradycardia, give further boluses of atropine until they disappear, and increase the infusion rate (Table 1).

Once the parameters have settled, see the patient at least hourly for the first 6 hours to check that the atropine infusion rate is sufficient and that there are no signs of atropine toxicity. As the required dose of atropine falls, observation for recurrence of cholinergic features can be done less often (every 2–3 hours). However, regular observation is still required to spot patients at risk of, and going into, respiratory failure.

## Atropine toxicity

Excess atropine causes agitation, confusion, urinary retention, hyperthermia, bowel ileus and tachycardia [15]. During regular observation for signs of overtreatment, check for the features given in Table 3.

The presence of all three suggests that too much atropine is being given. Stop the atropine infusion. Check again after 30 min to see whether the features of toxicity have settled. If not, continue to review every 30 min or so. When they do settle, restart at 70–80% of the previous rate. The patient should then be seen frequently to ensure that the new infusion rate has reduced the signs of atropine toxicity without permitting the reappearance of cholinergic signs.

Do not follow heart rate and pupil size because they can be fast or slow, and big or small, respectively, depending on the balance of nicotinic and muscarinic features. Tachycardia also occurs with rapid administration of oximes and with pneumonia, hypovolaemia, hypoxia, and alcohol withdrawal, and is not a contraindication to giving atropine.

Catheterise unconscious patients soon after resuscitation is completed. Look for urinary retention in an agitated confused patient; agitation may settle after insertion of the catheter.

## Care of the airway

If a pesticide-poisoned patient is unconscious, place an endotracheal (ET) tube at this point even if a Guedel airway is working well, to minimise the risk of aspiration and to facilitate respiratory care if there is deterioration.

Use diazepam to keep the patient sedated and tolerant of the ET tube. Because patients are often unstable during the first 6–12 hours, it may be better to sedate the patients to keep their ET tube in position if they start to waken with the atropine and the first dose of oxime.

## Active cooling and sedation

Hyperthermia is a serious complication in hot and humid wards. A febrile patient should receive the minimum amount of atropine needed to control muscarinic signs, sedation if there is excessive agitation and muscle activity, and active cooling. Lay a towel soaked with water over the patient's chest and place in a fan's airflow. Cold water soaked towels can also be placed at points of maximum heat loss (for example axillae, groins).

Reduce agitation with diazepam 10 mg given by slow IV push, repeated as necessary in an adult, up to 30–40 mg per 24 hours. Tying a non-sedated agitated patient to the bed is associated with complications, including death. Such patients struggle against their bonds and generate excess body heat, which may result in hyperthermic cardiac arrest.

Diazepam is preferred over haloperidol because large doses of haloperidol may be required in patients receiving atropine. Haloperidol is also non-sedating, associated with disturbances of central thermoregulation and prolongation of the QT interval, and pro-convulsant. Diazepam may also have other advantages because animal studies suggest that it reduces damage to the central nervous system [18] and diminishes central respiratory failure [8].

## Confirmation of exposure to cholinergic compounds

Confirmation of poisoning by anti-cholinesterase pesticides can be sought by measuring butyrylcholinesterase and/or red-cell acetylcholinesterase activity. However, such assays cannot be performed in the ward. Furthermore, emergency therapy should be determined by the patient's clinical features, not by knowledge of the ingested poison.

## Treatment of the resuscitated and stable patient – should gastric decontamination be performed?

Consider the need for gastric decontamination once the patient has been stabilised. Do not perform gastric decontamination until the patient is stable and, if necessary, intubated.

Ipecac is contraindicated in pesticide-poisoned patients. The effectiveness of both gastric lavage and activated charcoal is unknown.

### Gastric lavage

Consider lavage only if a patient has taken a highly toxic pesticide and arrives at hospital within 1–2 hours. It can be given to calm patients who have given explicit consent to the procedure or to unconscious intubated patients. Its use in agitated non-compliant patients or un-intubated drowsy or unconscious patients risks major complications including death.

Pass a nasogastric tube to decompress the stomach and to suck out its contents. If patients have been previously given forced emesis, their stomach may well be already filled with fluid.

If a decision is made to give lavage, after aspirating the stomach contents give water or normal saline in lots of 300 ml through a nasogastric tube. Larger volumes of fluid may push the poison into the small bowel. There is no reason to use a large-bore oro-gastric lavage tube for liquid poisons unless food blocks the nasogastric tube. Take off 300 ml before giving a further two or three 300 ml aliquots, otherwise the stomach may become distended, allowing fluid to pass into the small bowel or causing the patient to vomit. Measure the amount of fluid taken off to ensure that fluid is not left in the stomach.

### Activated charcoal

A dose of activated charcoal can be left in the stomach at the end of the lavage. There is currently no evidence that either single-dose or multiple-dose regimens of activated charcoal result in clinical benefit after pesticide poisoning.

## Oximes and other therapies

The clinical benefit of oximes for OP pesticide poisoning is not clear, being limited by the type of OP, poison load, time to start of therapy, and dose of oxime [19,20]. Current World Health Organisation guidelines recommend giving a 30 mg/kg loading dose of pralidoxime over 10–20 min, followed by a continuous infusion of 8–10 mg/kg per hour until clinical recovery (for example 12–24 hours after atropine is no longer required or the patient is extubated) or 7 days, whichever is later [20,21]. Where obidoxime is available, a loading dose of 250 mg is followed by an infusion giving 750 mg every 24 hours [20]. Too rapid administration will result in vomiting, tachycardia and hypertension (especially diastolic hypertension).

Oximes are not recommended for carbamate poisoning.

The role of hydrocortisone and antibiotic treatment after aspiration is not known. Aspiration of pesticide and stomach contents initially causes a chemical pneumonitis and not pneumonia [22]. It is unknown whether pneumonitis benefits from steroids. Pneumonia is diagnosed if the fever persists for more than 48 hours or there is focal consolidation on X-ray. Earlier use of antibiotics risks antibiotic-associated diarrhoea.

Alcohol co-ingestion requires assessment of blood sugar levels and vitamin B supplementation.

## Care after the first few hours

### General observation

OP/carbamate-poisoned patients are unstable and require regular observation to pick up changes in their general condition and their atropine requirements. Consider repeated doses of diazepam to keep the patient calm and settled.

If facilities permit, give patients a general anaesthetic, and intubate and mechanically ventilate them. This should reduce the number of deaths from respiratory complications.

### Observation for impending respiratory failure and recurring cholinergic crises

Watch for early signs of intermediate syndrome in OP-poisoned patients. Weakness of neck flexion is common: the patient has difficulty lifting their head off the pillow; subsequent signs include the use of accessory muscles of respiration, nasal flaring, tachypnoea, sweating, cranial nerve palsies and proximal muscle weakness in the limbs with retained distal muscle strength.

Not all patients with neck weakness will develop the full intermediate syndrome requiring intubation and ventilation, but such patients are at risk and should be seen regularly. Measure tidal or minute volume and blood gases, if available. A locally agreed value should act as a trigger for prophylactic sedation and intubation, followed as necessary by ventilation.

Recurrence of toxicity, requiring atropine therapy, commonly occurs after poisoning with fat-soluble OPs, such as fenthion, that leak out of fat over days and even weeks. Recurring cholinergic crises may occur with little notice.

## Conclusions

Medical management of severe cholinergic pesticide poisoning is difficult, with high mortality. Some patients will die no matter how well managed. However, careful resuscitation with appropriate use of antidotes, followed by good supportive care and observation, should minimise the number of deaths in the period after admission to hospital.

## Key messages

• 	Initial treatment of OP/carbamate pesticide poisoned patients involves the standard ABC of resuscitation.

• 	Since most deaths occur from respiratory failure, airway protection and ventilatory support is essential.

• 	Atropine can be given in an individualised dosing regimen to stabilise the patient.

• 	Careful observation probably saves many lives.

• 	Decontamination should only be done after the patient is fully stabilised, and not directly on admission.

## Competing interests

The authors declare that they have no competing interests.

## Abbreviations

ET = endotracheal; IV = intravascular; OP = organophosphorus.

## Supplementary Material

Additional file 1 Evidence for the protocol. Click here for file

## Figures and Tables

**Table 1:** An observation chart recording the initial atropinisation of an organophosphorus-poisoned patient

*Initials XX*	*Study number Axxxx*	*Date of arrival xx/xx/xx*								
Time	Heart rate >80	Clear lungs	Pupil size	Dry axilla	Syst. BP >80 mmHg	Bowel sounds (A/D/N/I)	Confused	Fever (>37.5°C)	Atropine infusion	Bolus given?
22.30	***52***	***Creps+***	***Pinpoint***	***No***	90/60	I	No	No		2.4 mg
22.35	***60***	***Creps+***	***Pinpoint***	***No***	90/60	I	No	No		4.8 mg
22.40	82	**+/-**	***Pinpoint***	Yes	110/60	N	No	No		4 mg
22.50	100	***Wheeze***	2 mm	Yes	-	D	No	No		2 mg
23.00	105	Clear	3 mm	Yes	-	D	No	No	2 mg/h	Infusion
23.15	105	Clear	3–4 mm	Yes	-	D	No	No	2 mg/h	Infusion
23.32	102	Clear	3–4 mm	Yes	-	D	No	No	2 mg/h	Infusion
00.30	98	Clear	3–4 mm	Yes	110/60	D	No	No	2 mg/h	Infusion
01.30	85	Clear	3–4 mm	Yes	-	D	No	No	2 mg/h	Infusion
02.30	***72***	***Wheeze***	3–4 mm	Yes	-	N/D	No	No		2 mg
02.35	96	Clear	3–4 mm	Yes	-	D	No	No	2.4 mg/h	Infusion
02.45	98	Clear	3–4 mm	Yes	-	D	No	No	2.4 mg/h	Infusion
04.00	102	Clear	3–4 mm	Yes	-	D	No	No	2.4 mg/h	Infusion

Atropinisation was reached at 23.00, 30 min after the first atropine dose was given; a total of 13.4 mg of atropine was required. After 10 min, doubling doses were no longer used because there was a clear response to therapy with the pulse climbing above 80 beats/min and the chest sounding better. After a further 1.5 hours, the pulse rate started to drop but it was not until it had dropped below 80 beats/min and wheeze had become audible in the chest that another 2 mg bolus was given to atropinise the patient again. The atropine infusion rate was also increased and the patient remained stable for the next few hours.

**A/D/N/I, absent/decreased/normal/increased**; creps, crepitations; syst. BP, systolic blood pressure. **Clinical features in bold type indicate that atropine is required. Dashes indicate that no BP reading was taken.**

**Table 2:** Target end-points for atropine therapy

Clear chest on auscultation with no wheeze
Heart rate >80 beats/min
Pupils no longer pinpoint
Dry axillae
Systolic blood pressure >80 mmHg

Notes:

1. The aim of atropine therapy is to clear the chest and reach the end-points for all five parameters.

2. There is no need to aim for a heart rate of 120–140 beats/min. This suggests atropine toxicity rather than simple reversal of cholinergic poisoning. Such high heart rates will cause particularly severe complications in older patients with pre-existing cardiac disease – myocardial infarctions may result. However, tachycardias are also caused by hypoxia, agitation, alcohol withdrawal, pneumonia, hypovolaemia, and fast oxime administration. Tachycardias are not a contraindication for atropine if other features suggest under-atropinisation.

3. Aspiration will commonly result in focal crepitations. Attempt to distinguish such crepitations from the more general crepitations of bronchorrhoea.

4. Splashes of organophosphorus into the eye will produce intense miosis that may not respond to atropine therapy. However, symmetrical miosis is likely to be due to systemic effects of the ingested pesticide.

**Table 3:** Markers used to assess atropine toxicity

Confusion
Pyrexia
Absent bowel sounds (Urinary retention)

Notes:

Many factors can cause confusion and pyrexia. However, confusion and/or pyrexia in the absence of bowel sounds suggests that they are due to atropine toxicity and will respond to a reduction in the rate of atropine administration.

Alcohol withdrawal, requiring benzodiazepine therapy, must be considered in poisoned patients who are confused.

Control pyrexia as soon as possible; conditions causing pyrexia include agitation from alcohol withdrawal or atropine toxicity, atropine-induced failure to sweat, and high ambient temperature. Active cooling of the patient with fan and water-soaked towels must be a priority because they are at risk of hyperthermia-induced cardiac arrest. Most ill patients will be catheterised after resuscitation to observe urinary output. Urinary retention can therefore not then be used as a marker of toxicity.
