# Supplementary material for: Early management after self-poisoning with an organophosphorus or carbamate pesticide – a treatment protocol for junior doctors
Source: Crit Care. 2004 Sep 22;8(6):R391–7. doi: 10.1186/cc2953 (PMC1065055; doi:10.1186/cc2953)
Supplement: Additional file 1 — Evidence for the protocol. [file cc2953-S1.pdf]

Early Management of an Unconscious Patient after Self-Poisoning with an Organophosphorus or Carbamate Pesticide

Eddleston et al.

Appendix - Evidence for the protocol.

The accompanying paper lays out guidelines for managing severely ill pesticide poisoned patients. Unfortunately, despite the large number of pesticide poisoning cases occurring worldwide every year, the current evidence base is small [1].

The evidence used for the protocol comes from systematic reviews of the literature [2,3] and the treatment of over 3000 pesticide poisoned patients in a RCT in Sri Lankan district hospitals. The guidelines have been developed over the last two years and are used routinely by doctors who have just finished medical school, before they begin their internship.

Initial assessment of the poisoned patient

This follows standard practice with preservation of the airway, provision of oxygen and resuscitation.

Use of atropine

Since the only life-saving antidotes for pesticide poisoning are oxygen and atropine, and oxygen has already been given, the most important issue after resuscitation is to decide whether the patient has taken a cholinergic pesticide and requires atropine. The clinical features of cholinergic poisoning that should trigger the decision to give atropine have been well described in multiple case series [4].

The best regimen for the administration of atropine has not been established [5]. A study performed in Bangalore, India, found that a regimen of bolus loading doses followed by an infusion improved outcome compared to repeated bolus doses [6] (note, however, that the study used historical controls which risks inflating benefit compared to RCTs [7,8]). Although the benefit of infusions is not yet proven, the use of bolus loading doses followed by an infusion may save time, require less observation, produce less fluctuation in plasma atropine concentrations, and make weaning easier [5].

We prefer to give low doses of atropine to start with and then rapidly escalate the dose. An alternative approach is to start with much bigger doses, to ensure rapid atropinisation, and then wait for the atropine levels to fall. Because of the dangers of over-atropinisation, however, the former practice offer more control by starting with low doses. We have been unable to distinguish patients who needed very large doses of atropine from those who required just a few milligrams.

The initial half life of distribution of atropine is about 1 minute [5,9]. Studies in anesthetized patients indicate that the peak effect is seen within three minutes of an IV injection [11]. There is therefore no need to wait for more than five minutes before checking for a response and giving another bolus dose if no response has occurred.

Giving atropine before oxygen

Many textbooks state that atropine should not be given to a cyanosed patient until oxygen has been given - to reduce the risk of atropine inducing ventricular tachy-cardias. While apparently sensible, such advice risks preventing doctors working in

small rural hospitals from giving life-saving atropine treatment, since many do not have oxygen. Furthermore, in our treatment of more than 800 patients receiving atropine, many of whom received atropine before oxygen, no patient had a cardiac arrest within minutes of giving atropine (Eddleston, unpublished).

The primary evidence for an increased risk of a ventricular dysrhythmia from giving atropine to a cyanosed patient consists of very few patients. Since atropine dries secretions and reduces bronchospasm, its administration should reduce cyanosis.

Giving fluids

There have been no studies on the effects of giving IV fluids in ill patients with OP poisoning. However, due to the cholinergic effects, these patients lose a great deal of fluid into their gastrointestinal tract and lungs, and onto their skin as sweat, resulting in intravascular fluid depletion. Some also develop a severe diarrhoea that results in fluid and potassium loss.

There is no evidence that giving fast IV fluid to patients with bronchorrhoea is dangerous as long as atropine is being given simultaneously to dry the lung secretions.

Criteria for atropinisation

There are no comparative studies of markers for adequate atropinisation [5]. However, patients die acutely from respiratory or circulatory failure, the former of which is exacerbated by bronchospasm and bronchorrhoea. All respond to atropine treatment. Therefore, in our study, we use air entry on chest auscultation, heart rate, and blood pressure as the main parameters for adequate atropinisation.

Atropine toxicity

Patients can be over-atropinised to reduce the risk of them becoming under-atropinised. However, atropine toxicity has its risks and careful observation of patients reduces the need for giving so much atropine. Hyperthermia is a particularly serious complication in the hot wards of the developing world: hyperthermia resulting from the high ambient temperatures is exacerbated by intense muscle activity due to atropine-induced agitation and failure of sweating, and alcohol withdrawal.

Observation and sedation of patients

Pesticide-poisoned patients are at high risk of lapsing into unconsciousness and developing respiratory failure. There is no evidence that diazepam, if given slowly and carefully in doses up to 40mg in an adult, causes respiratory failure. Do not hold back diazepam simply because pesticide-poisoned patients **may** develop respiratory failure – early intubation is easy in a sedated patient, as is giving other therapies that may be required.

Gastric decontamination

Ipecacuanha takes 20-30min to work and vomiting may last for more 30min [11]. If loss of consciousness occurs during this time, intubation may be needed in an unconscious vomiting patient. Since patients with pesticide poisoning may suddenly deteriorate, ipecacuanha is contraindicated. Forced emesis in hospital using other techniques is ineffective [11].

The efficacy of gastric lavage falls rapidly with time since ingestion [12]. By the time most patients arrive in hospital, the majority of pesticide will have passed into the small bowel, out of the reach of gastric lavage. Some diluted solvent may be left in the stomach – this will smell of ‘pesticide’ if sucked out with a NG tube. The volume of fluid in the stomach will appear large in cholinergic poisoning due to the secretion of fluid into the bowel [4].

There is currently no evidence that either single or multiple dose regimens of activated charcoal result in clinical benefit [13,14]. The practice of giving charcoal rests upon usual practice and is now being evaluated in an RCT (ISRCTN02920054).

Oximes

Carbamate-inhibited AChE regenerates spontaneously faster than OP-inhibited AChE [4]. As a result, oximes are not recommended for carbamate poisoning [4]. Although there are suggestions from animal studies that oximes may make carbamate poisoning worse, there is no evidence of harm in humans and it seems sensible to give oximes to symptomatic patients who have ingested an unknown cholinergic pesticide. However, some authors recommend giving oximes for all significant carbamate poisonings [15].

References

1. NA Buckley, L Karalliedde, A Dawson, N Senanayake, M Eddleston: **Where is the evidence for the management of pesticide poisoning - is clinical toxicology fiddling while the developing world burns?** *J Toxicol Clin Toxicol* 2004, **42:** 113-116.

2. M Eddleston, S Singh, N Buckley: **Acute organophosphorus poisoning.** *Clinical Evidence* 2003, **10:** 1652-1663.

3. M Eddleston, L Szinicz, P Eyer, N Buckley: **Oximes in acute organophosphorus pesticide poisoning: a systematic review of clinical trials.** *Q J Med* 2002, **95:** 275-283.

4. B Ballantyne, TC Marrs: **Overview of the biological and clinical aspects of organophosphates and carbamates.** In *Clinical and experimental toxicology of organophosphates and carbamates*. Edited by Ballantyne B, Marrs TC. Oxford: Butterworth heinemann; 1992:3-14.

5. International Programme on Chemical Safety. Antidotes for poisoning by organophosphorus pesticides. Monograph on atropine. http://www.intox.org/databank/documents/antidote/antidote/atropine.htm . 2002.

6. J Sunder Ram, SS Kumar, A Jayarajan, G Kuppuswamy: **Continuous infusion of high doses of atropine in the management of organophosphorus compound poisoning.** *J Assoc Physicians India* 1991, **39:** 190-193.

7. KF Schulz, I Chalmers, RJ Hayes, DG Altman: **Empirical evidence of bias: dimensions of methodological quality associated with estimates of treatment effects in controlled trials.** *JAMA* 1995, **273:** 408-412.

8. R Kunz, AD Oxman: **The unpredictability paradox: review of empirical comparisons of randomised and nonrandomised clinical trials.** *BMJ* 1998, **317:** 1185-1190.

9. AJW Heath, T Meredith: **Atropine in the management of anticholinesterase poisoning.** In *Clinical and experimental toxicology of organophosphates and carbamates*. Edited by Ballantyne B, Marrs T. Oxford: Butterworth Heinemann; 1992:543-554.

10. RK Mirakhur, CJ Jones, JW Dundee: **Effects of intravenous administration of glycopyrrolate and atropine in anaesthetised patients.** *Anaesthesia* 1980, **35:** 277-281.

11. American Academy of Clinical Toxicology and European Association of Poisons Centres and Clinical Toxicologists: **Position statement: ipecac syrup.** *J Toxicol Clin Toxicol* 1997, **35:** 699-709.

12. American Academy of Clinical Toxicology and European Association of Poisons Centres and Clinical Toxicologists: **Position statement: gastric lavage.** *J Toxicol Clin Toxicol* 1997, **35:** 711-719.

13. American Academy of Clinical Toxicology and European Association of Poison Centres and Clinical Toxicologists: **Position statement: single-dose activated charcoal.** *J Toxicol Clin Toxicol* 1997, **35:** 721-741.

14. American Academy of Clinical Toxicology and European Association of Poison Centres and Clinical Toxicologists: **Position statement and practice guidelines on the use of multi-dose activated charcoal in the treatment of acute poisoning.** *J Toxicol Clin Toxicol* 1999, **37:** 731-751.

15. CK Aaron: **Organophosphates and carbamates.** In *Clinical management of poisoning and drug overdose*. Edited by Haddad LM, Shannon MW, Winchester JF. W.B.Saunders; 2004.
